# Extramedullary haematopoiesis presented as intrathoracic tumour in a patient with alpha-thalassaemia

**DOI:** 10.1186/1749-8090-8-120

**Published:** 2013-05-01

**Authors:** Dmitry Bobylev, Ruoyu Zhang, Axel Haverich, Marcus Krueger

**Affiliations:** 1 Department of Cardiothoracic, Transplantation, and Vascular Surgery, Hannover Medical School, Carl-Neuberg-Str. 1, 30625, Hannover, Germany

**Keywords:** Extramedullary haematopoiesis, Alpha-thalassemia, Tumour-like masses

## Abstract

The authors report a case of extramedullary haematopoiesis (EMH) presenting as an intrathoracic tumour in a patient with alpha-thalassaemia. CT scan and MRI of the chest were obtained and followed by tumour excision. Compared to beta-thalassaemia, only two cases of EMH in patients with alpha-thalassaemia have been described in the literature. A possible reason for this disparity is discussed.

## Background

Extramedullary haematopoiesis (EMH) is a compensatory phenomenon in hematological diseases with insufficient blood cell production, either due to a bone marrow replacement disease (e.g. myelofibrosis) or hemolytic anaemia with ineffective erythropoesis (e.g. betathalassaemia, sickle cell anaemia, and hereditary spherocytosis). Only a few patients with alpha-thalassaemia have been reported to present EMH [1,2].

## Case presentation

A 42-year-old female immigrant from Vietnam with known alpha-thalassaemia (Hb H disease) was admitted to the Department of Neurosurgery of our hospital due to persistent back pain, lumbago and intermittent pain in the right lower limb. Upon admission her haemogram showed a haemoglobin level of 9.6 g/dl and a mean corpuscular volume of 69.8 fl. The past medical history included a splenectomy in 2006 and repeated blood transfusions within the last 4 years. In 1998, a examination of her Hb H disease revealed numerous Hb H cells with Brilliant Cresyl Blue staining, 16.8% Hb H, 2.5% Hb A2 and 2.7% Hb F by Haemoglobin electrophoresis, as well as a α°/α+α-genotype with α --SEA deletion on one chromosome. The requisite point mutation on one allele of the other chromosome remains unknown.

**Figure 1:**
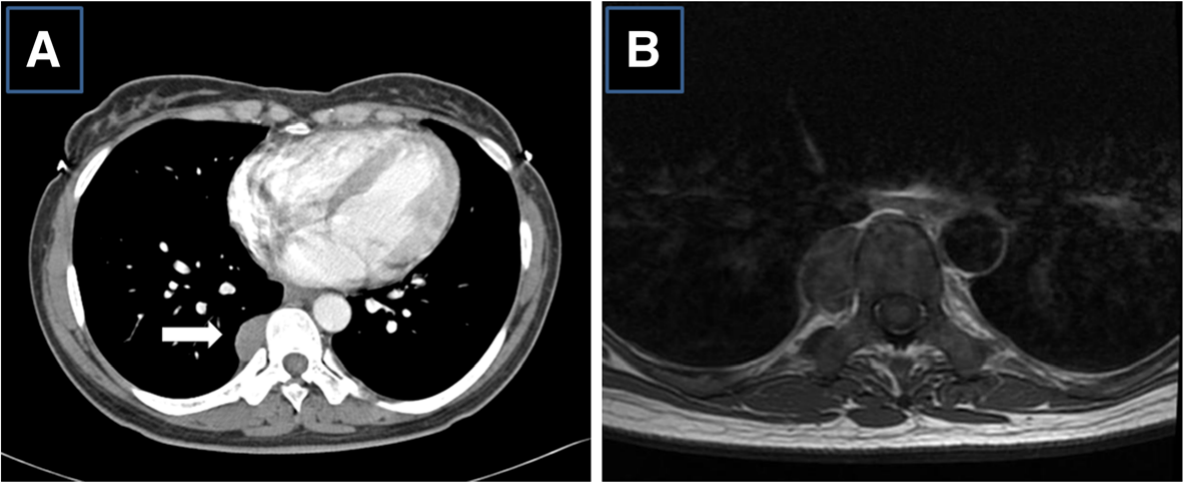
**Preoperative images. A**) CT scan of the chest demonstrated a paravertebral soft tissue mass (white arrow) through the region Th7 – Th9. **B**) MRI of the chest could not exclude an invasion into the spinal canal.

**Figure 2:**
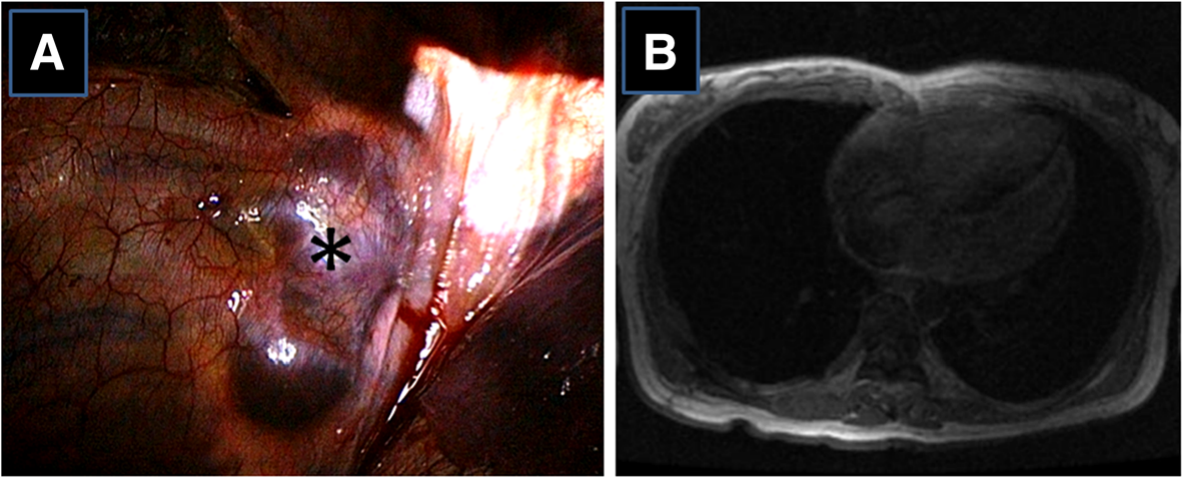
**Intraoperative photo and postoperative image. A**) Intraoperative photo showing a well-demarcated, dark-coloured mass (* = extramedullary haematopoietic tumour). **B**) MRI of the chest, obtained 7 months after surgery, revealed no local recurrence.

A CT scan of the chest obtained in the referring hospital showed a paravertebral, ovoid smooth tissue mass in the right thoracic cavity. This mass was 6.6 cm × 3.3 cm in size, reaching from Th7 to Th9 (Figure 1A). An MRI of the chest could not rule out a tumour invasion into the spinal canal (Figure 1B). The patient was subjected to a video-assisted thoracoscopy that revealed a well-demarcated, dark-coloured tumour in the right thoracic cavity, without infiltration of the lung (Figure 2A). Thus, a posterolateral thoracotomy with careful dissection of the tumour mass was performed along with the preservation of intercostal blood vessels and nerves. Invasion into the intervertebral foramen was not observed and the mass could be resected completely. The histological examination of the mass revealed EMH. The postoperative course was uneventful and an MRI of the chest, obtained 7 months after surgery, revealed no local recurrence (Figure 2B).

## Discussion

EMH is a compensatory mechanism due to bone marrow dysfunction. This haematopoetic response is most often microscopic, but can also result in organomegaly or the development of tumour-like masses, usually affecting the liver, spleen, and lymph nodes, while the intrathoracic cavity is less frequently involved [3,4].

Intrathoracic EMH masses are generally located in the posterior mediastinum but can also manifest as interstitial pulmonary abnormality, pleural mass or haemothorax, either alone or in combination. EMH can be single or multiple and may present bilaterally. As it is mostly an asymptomatic finding, intrathoracic EMH is usually detected incidentally on chest radiographs. The diagnosis can be established with percutaneous fine-needle aspiration or thoracoscopic biopsy. EMH usually regresses or disappears after treatment with blood transfusions and hydroxyurea. Radiation therapy or surgical intervention can be required in case of clinical symptoms, especially if spinal cord compression occurs [5-7], or in case of rapid growth which can lead to an increased risk of rupture and consecutive haemothorax.

Only two cases of alpha-thalassaemia (Hb H disease) associated with intrathoracic EMH have been previously reported. Wu et al. described the case of intrathoracic EMH in a 34-year-old male who initially presented with nonspecific symptoms, extreme hyperbilirubinaemia and multiple intrathoracic bilateral paravertebral tumours [1]. In the report of Chu et al., a 44-year-old patient developed dyspnea and a massive left-side haemothorax. A CT scan and MRI of the chest showed multiple paravertebral masses, which were found to be EMH by thoracoscopic biopsy [2].

In general, haemolysis, rather than ineffective erythropoiesis, is the primary cause of anaemia in Hb H disease and, therefore, EMH is a very rare complication. However, so-called “non-deletional Hb H” (with a point-mutation in the third allele) is often more severe and likely to require transfusions, as the non-functional alpha globin chain of the haemoglobin molecule can bind to the red blood cell membrane, causing an increased rate of both haemolysis and ineffective erythropoiesis (with erythroid apoptosis in the bone marrow) [8].

## Conclusion

This case report suggests that EMH should be considered in the differential diagnosis in alpha-thalassaemia patients with an intrathoracic mass.

## Consent

Written informed consent was obtained from the patient for publication of this case report and accompanying images. A copy of the written consent is available for review by the Editor-in-Chief of this journal.

## Abbreviations

g/dl: Grams per decilitre; fl: Femtolitre; Hb: Haemoglobin; Hb A: Adult haemoglobin; Hb F: Fetal haemoglobin; α -- SEA: Southeast Asian type; Th: thoracic vertebrae.

## Competing interests

The authors declare that they have no competing interests.

## Authors’ contributions

All authors have no financial or other interests regarding the submitted manuscript. DB conceived the study, wrote the manuscript, RZ participated in the coordination of this study, provided the information of the patient, performed literature search, AH supervised and reviewed the manuscript, MK was the operating surgeon of the patient, supervised and reviewed the manuscript. All authors read and approved the final manuscript.
